# Cardiovascular disease risk factors among older people: Data from the National Health and Morbidity Survey 2015

**DOI:** 10.1371/journal.pone.0240826

**Published:** 2020-10-21

**Authors:** Shariff Ghazali Sazlina, Rajini Sooryanarayana, Bee Kiau Ho, Mohd. Azahadi Omar, Ambigga Devi Krishnapillai, Noorlaili Mohd Tohit, Sheleaswani Inche Zainal Abidin, Suthahar Ariaratnam, Noor Ani Ahmad

**Affiliations:** 1 Department of Family Medicine, Faculty of Medicine and Health Sciences, Universiti Putra Malaysia, Serdang, Selangor, Malaysia; 2 Malaysian Research Institute on Ageing (MyAgeing^™^), Universiti Putra Malaysia, Serdang, Selangor, Malaysia; 3 Family Health Development Division, Ministry of Health Malaysia, Putrajaya, Wilayah Persekutuan Putrajaya, Malaysia; 4 Klinik Kesihatan Bandar Botanik, Ministry of Health Malaysia, Bandar Botanic, Klang, Selangor, Malaysia; 5 National Institutes of Health, Ministry of Health Malaysia, Setia Alam, Shah Alam, Malaysia; 6 Department of Family Medicine, Faculty of Medicine and Health, National Defense University of Malaysia, Kuala Lumpur, Malaysia; 7 Department of Family Medicine, Faculty of Medicine, National University of Malaysia, Cheras, Kuala Lumpur, Malaysia; 8 Elderly Health Sector, Family Health Development Division, Ministry of Health, Putrajaya, Wilayah Persekutuan Putrajaya, Malaysia; 9 Department of Psychiatry, Faculty of Medicine, Universiti Teknologi MARA (UiTM), Selayang Campus, Batu Caves, Selangor, Malaysia; 10 Centre for Family Health Research, Institute for Public Health, National Institutes of Health, Ministry of Health Malaysia, Setia Alam, Shah Alam, Selangor, Malaysia; University of Mississippi Medical Center, UNITED STATES

## Abstract

Study on cardiovascular disease (CVD) risk factors and their prevalence among the older people in Malaysia is limited. We aimed to determine the prevalence and factors associated with CVD risk factors using the non-laboratory Framingham Generalized 10-Year CVD risk score among older people in Malaysia. This was a population-based cross-sectional study using data of 3,375 participants aged ≥60 years from the National Health and Morbidity Survey 2015. Sociodemographic, health factors and clinical assessments (anthropometry and blood pressure) were included. Complex survey analysis was used to obtain prevalence with 95% confidence intervals (CI). We applied ordinal regression to determine the factors associated with CVD risk. The prevalence for the high 10-year CVD risk was 72.1%. Body mass index was higher among those aged 60–69 years in men (25.4kg/m^2^, 95%CI 25.1–25.8) and women (26.7kg/m^2^, 95%CI 26.3–27.1) than the other age groups. The factors associated with moderate and high 10-year CVD risk were Malay ethnicity (Odds Ratio(OR) 0.76, 95%CI 0.63–0.92, p = 0.004), unmarried status (OR 1.55, 95%CI 1.22–1.97, p<0.001) and physically inactive (OR 0.72, 95%CI 0.55–0.95, p = 0.020). There is a need for future study to evaluate preventive strategies to improve the health of older people in order to promote healthy ageing.

## Introduction

Cardiovascular disease (CVD) is the leading cause of mortality globally and it is known that CVD increases with age [1]. According to the World Health Organization (2017), 17.7 million people die annually from CVD primarily due to coronary artery disease and stroke, which accounted for 31% of all global deaths. It is associated with tobacco use, unhealthy diet, physical inactivity and sedentary behaviour, which are reflected by the increased prevalence of hypertension, diabetes, overweight and obesity. The prevalence of these non-communicable diseases increase with age, which leads to significant mortality among older people as well as disability, functional decline and healthcare costs [2].

In Malaysia, the leading cause of CVD death in 2016 was coronary artery disease at 13.2% followed by stroke at 6.9% [3]. The percentage of deaths due to CVD in the public hospitals have increased from 15% in 2006 to 25.4% in 2010 [4]. Majority of these are among people aged ≥60 years. The National Health and Morbidity Surveys (NHMS) has shown that the prevalence of CVD risk factors such as hypertension, dyslipidemia, diabetes, overweight/obesity and smoking has been on an increasing trend and it increases with age [5]. This has led to increased demand for healthcare, especially with a high proportion of older age groups [6].

Malaysia is experiencing a demographic transition due to an increasing aged population ≥60 years and increased life expectancy [7]. The longevity of the older population has raised the requirement of health care services due to the increase in prevalence of chronic diseases and disability. Ageing brings along an uneven increase in CVD with the related disabilities [8]. The ability of older people to remain healthy and independent requires the provision of appropriate health care to meet their needs.

In order to develop and implement an effective strategy for prevention and treatment of CVD in older people, it is critical to have a more comprehensive understanding of a wide range of CVD risk factors and the factors salient to this population. However, few studies focused on the older people [9, 10]. Once significant CVD risk factors and their prevalence are identified among community dwelling older people, researchers and clinicians will be able to develop and implement effective intervention strategies to ensure healthy and productive ageing of the Malaysian population.

The association of biologic risk factors such as hypertension, diabetes, and dyslipidaemia with CVD has been studied in developed countries [11, 12]. In addition to biologic risk factors, many epidemiologic studies have demonstrated positive associations between alcohol intake, smoking, physical inactivity, and obesity with the prevalence of CVD [13, 14]. However, these studies focused on young to middle age groups and were from developed countries. The Framingham Generalized 10-Year CVD Risk Score (FRS) is a validated commonly used tool to quantify the CVD risk [15]. Studies on prevalence and risk factors for CVD among older people in Malaysia is limited. Therefore, we aimed to determine the prevalence of CVD risk factors and the associated factors of cardiovascular risk using the non-laboratory FRS among community dwelling older people in Malaysia.

## Methods

### Data source

This cross-sectional study used data from the National Health and Morbidity Survey 2015 (NHMS 2015) conducted by the Institute for Public Health, National Institutes of Health and funded by Ministry of Health, Malaysia. The Ministry of Health Medical Research Ethics Committee approved the study and the detailed description of the sampling methods are as described in the NHMS 2015 Methodology & General Findings report [16]. Briefly, the NHMS 2015 involved Malaysian residents in non-institutionalised living quarters in both urban and rural from all 13 states and 3 Federal Territories (FTs) in Malaysia. The Department of Statistics Malaysia performed the sample selection using the sampling frame that comprised 79,240 geographical areas known as Enumeration Blocks (EB) with 7.65 million living quarters (LQs). On average, each EB comprised 80 to 120 LQ with 500 to 600 people. A two-stage stratified random sampling was employed for national representativeness. The states and FTs were considered as Primary Stratum, while urban and rural areas within the states were Secondary Stratum. All states and FTs were included in this survey. The EBs from urban and rural areas in each states and FTs were randomly selected. The sample selection involved two stages: 1) selection of EBs (536 EBs and 333 EBs from urban areas and rural areas, respectively), which were considered as Primary Sampling Unit, and 2) selection of LQs within the EBs, which were considered as Secondary Sampling Unit. In each EB, a total of 12 LQs were randomly selected and all households and individuals within the selected LQs were invited to participate in this survey. Random selection of EBs and LQs was done by the Department of Statistics Malaysia. In the present study, data of participants aged 60 years was extracted and analysed.

### Sample size

The NHMS 2015 dataset comprised 20,747 participants aged ≥18 years. We extracted a total of 3,790 data of participants aged ≥60 years from the NHMS 2015 dataset. However, only 3,375 had complete data for the calculation of FRS and were included in the analysis of this study.

### Variables

CVD risk was the main outcome of interest in this study. The Framingham Generalised 10-Year CVD Risk Score (FRS) evaluates the actual risk of CVD and the risk estimates are age, HDL cholesterol, total cholesterol, systolic blood pressure (BP), smoking, and diabetes [15]. In our study, the calculation of FRS was based on the algorithm developed by D’Agostino et al. (2008) and the risk estimates are the same for all parameters except this algorithm used non-laboratory based methods where they substituted BMI for cholesterol [17]. Scores were summed separately for men and women in view of the differences in risks, and the FRS were categorised as low (10-year risk of <10%), moderate (10-year risk of 10–20%) (moderate) or high (10-year risk of >20%) risk.

The independent variables used for analysis in this study included socio-demography, health-related factors and clinical assessments. These variables were extracted from the NHMS 2015 dataset and the tools used to collect these data has been described in the NHMS 2015: Methodology & General Findings report [16]. The socio-demography included age, gender, ethnicity, marital status, location: rural or urban, highest level of education and household monthly income. The health-related factors included in this study were known history of diabetes, presence of hypertension and history of treatment over the past two weeks, presence of dyslipidemia and lifestyle information (physical activity, smoking status). The physical activity was measured using the short version of International Physical Activity Questionnaire (IPAQ) on walking, moderate-intensity activities, vigorous-intensity activities and sitting duration over the last 7 days [18]. The physical activity was categorised into three categories: inactive, minimally active and health-enhancing physical activity (HEPA).

The clinical assessments included the anthropometric measurements (BMI and waist circumference) and BP measurements. The weight was measured in kilograms (kg) using the Tanita Personal Scale HD 319 to the nearest two decimal point and height was measured with the SECA Stadiometer 213 in metres. The BMI was calculated based on a formula of weight (in kg) divided by square of height (in metre). The Omron Japan Model HEM-907, which had been validated and calibrated was used for blood pressure assessment. Waist circumference was measured and categorised as abnormal if ≥80cm in women and ≥90cm in men [19]. All the clinical assessments were conducted by trained nurses during the NHMS 2015 survey.

### Ethical approval

This study was approved by the Medical Research Ethics Committee, Ministry of Health, Malaysia [NMRR-14-1064-21877]. Both verbal and written consent were obtained from the participants. Participation was voluntary. The participants were assigned non-identifiable identification codes for data entry and data analysis. The participants would not be identified in the report writing or publication.

### Data analysis

Complex survey analysis was used to obtain prevalence and population estimates with 95% confidence intervals. To improve the representativeness of the sample in terms of the size, distribution, and characteristics of the study population, sample weights were calculated for each respondent prior to the analysis. The basic weight for each sampled household would be equal to the inverse of its probability of selection (calculated by multiplying the probabilities at each sampling stage). The basic weight was adjusted based on the non-response and post-stratification factor to derive the sample weights.

Descriptive analysis was performed, where continuous data was presented as mean with standard deviation considering the dataset was large and normality was assumed. Categorical data were presented in frequency and column percentage For the purpose of analysis, three categories of age groups were described: (i) aged 60–69 years; (ii) aged 70–79 years; and (iii) aged 80 years and above.

Univariate analysis was carried out using Pearson’s Chi-square test, independent sample *t*-test and one-way ANOVA. A Chi-square test was applied to determine the proportion of achieving target controls for CVD risks across age groups. For the CVD risk, we applied ordinal regression using the logit model to determine the factors associated with CVD risk based on the FRS 10-year risk since the FRS risk was categories into 3 ordinal categories (i.e.: 10-year risk of <10%, 10-year risk of 10–20% and 10-year risk of >20%) with the 10-year risk of <10% was used as the reference group. We calculated the cumulative odds ratio from the parameter estimates obtained from the logit model. The cumulative odds ratio (OR) with 95% confidence interval (95%CI) and *P*-value were presented. We did not include age, gender, BP, BMI, smoking status and history of hypertension treatment, diabetes and dyslipidemia because these were considered in the calculation of the FRS. All analyses were carried out using SPSS version 24.0 (IBM, Armonk, NY, USA).

## Results

### Characteristics of participants

The mean age of the participants was 68.1 years with the majority in the age group 60–69 years (64.7%). Majority of the participants were women (50.8%), Malay ethnicity (47.8%), with highest primary school education (68.0%), married (69.3%), and lived in the rural areas (28.2%). The mean monthly household income was RM3007 (95%CI = 2748, 3267). Table 1 summarises the characteristics of the study participants.

**Table 1:** Characteristics of study participants.

Characteristics	% (95%CI)	Mean (95%CI)
**Age (N = 3375)**		
• 60–69 years	64.7 (62.1–67.3)	
• 70–79 years	28.1 (25.8–30.5)	
• ≥80 years	7.2 (5.9–8.7)	
**Sex (N = 3375)**		
• Men	49.2 (47.3–51.0)	
• Women	50.8 (49.0–52.7)	
**Ethnicity (N = 3375)**		
• Malay	47.8 (43.5–52.1)	
• Chinese	35.1 (31.1–39.3)	
• Indian	6.8 (5.4–8.6)	
• Other *Bumiputera*	9.0 (6.7–12.1)	
• Others	1.3 (0.8–1.9)	
**Highest level of education (N = 3348)**		
• No formal education	19.8 (17.6–22.3)	
• Primary school	48.2 (45.5–51.0)	
• Secondary school and higher	32.0 (29.3–34.7)	
**Marital status (N = 3375)**		
• Married	69.3 (66.9–71.5)	
• Unmarried	30.7 (28.5–33.1)	
**Strata (N = 3375)**		
• Urban	71.8 (69.1–74.3)	
• Rural	28.2 (25.7–30.9)	
**Monthly household income, RM (N = 3375)**		3007 (2748–3267)
**Presence of diabetes (N = 3375)**	38.7 (36.2–41.2)	
**Presence of hypertension (N = 3375)**	70.4 (68.3–72.5)	
**Presence of dyslipidaemia (N = 3375)**	64.8 (62.4–67.1)	
**Systolic BP, mmHg (N = 3375)**		143.5 (142.5–144.5)
**Diastolic BP, mmHg (N = 3375)**		79.2 (78.6–79.8)
**On antihypertension medications (N = 823)**		
• Yes	87.4 (84.0–90.1)	
• No	12.6 (9.9–16.0)	
**Smoking status (N = 3375)**		
• Current smoker	12.8 (11.3–14.4)	
• Never smoked	82.9 (81.1–84.5)	
• Former smoker	4.4 (3.5–5.4)	
**BMI, kg/m**^**2**^ **(N = 3375)**		25.4 (25.2–25.6)
**Waist circumference (N = 3367)**		
• Abnormal (Men ≥90cm, Women ≥80cm)	62.9 (60.3–65.4)	
• Normal (Men <90cm, Women <80cm)	37.1 (34.6–39.7)	
**Physical activity (N = 3367)**		
• Inactive	45.2 (42.9–47.6)	
• Minimally active	35.0 (32.8–37.2)	
• HEPA active	19.8 (18.2–21.5)	
**FRS (N = 3375)**		
• Low 10-year CVD risk	7.2 (6.1–8.5)	
• Moderate 10-year CVD risk	20.7 (19.0–22.5)	
• High 10-year CVD risk	72.1 (70.1–74.0)	

95%CI = 95% confidence interval, SD = standard deviation, BMI = body mass index, FRS = framingham risk score, CVD = cardiovascular disease

The calculated FRS showed that 72.1% had high 10-year CVD risk. The prevalence of hypertension was 70.4%, of whom 87.4% were on antihypertension medications. The mean systolic and diastolic blood pressure were 143.5mmHg and 79.2mmHg, respectively. About a third had diabetes (38.7%) and two thirds had dyslipidaemia (64.8%); while majority of them never smoked (82.9%) and had abnormal waist circumference (62.9%). The mean BMI was 25.4kg/m^2^ and 45.2% were inactive.

The CVD risk factors were compared between the different age groups as shown in Table 2. The analysis separates those for men and women due to differences in risks. We found that women had significantly higher mean systolic BP (145.2mmHg, 95%CI 143.8–146.6) and higher proportion of dyslipidemia (73.6%, 95%CI 70.5–76.4) than men (mean systolic BP: 141.7mmHg, 95%CI 140.4–143.1; dyslipidemia: 55.7%, 95%CI 52.2–59.1). Compared to the other age groups, the BMI was significantly higher among those aged 60–69 years in men (25.4kg/m^2^, 95%CI 25.1–25.8) and women (26.7kg/m^2^, 95%CI 26.3–27.1). However, there were no significant difference between the age groups on the other CVD risk factors for men and women.

**Table 2:** Cardiovascular disease risk factors according to age group by gender (N = 3375).

Group	Presence of diabetes, % (95%CI)	Presence of dyslipidemia, % (95%CI)	Systolic BP, mmHg Mean (95%CI)	Current smoking, % (95%CI)	BMI, kg/m^2^ Mean (95%CI)
**Total**	38.7 (36.2,41.2)	64.8 (62.4,67.1)	143.5 (142.5,144.5)	12.8 (11.3,14.4)	25.4 (25.2,25.6)
**Men**					
Overall	36.6 (33.3,40.0)	55.7 (52.2,59.1)	141.7 (140.4,143.1)	24.7 (21.9,27.8)	24.9 (24.6,25.2)
Age					
• 60–69	37.4 (33.4,41.5)	55.3 (50.9,59.6)	141.5 (140.0,143.0)	27.0 (23.6,30.8)	25.4 (25.1,25.8)
• 70–79	34.9 (29.0,41.3)	57.7 (51.1,64.0)	141.8 (139.2,144.4)	20.7 (15.9,26.5)	24.1 (23.7,24.6)
• ≥ 80	36.0 (23.8,50.3)	50.2 (36.2,64.2)	144.0 (137.3,150.6)	18.0 (9.8,30.6)	22.1 (21.1,23.1)
**Women**					
Overall	40.7 (37.3,44.2)	73.6 (70.5,76.4)	145.2 (143.8,146.6)	1.2 (0.7,2.0)	25.9 (25.6,26.2)
Age					
• 60–69	39.9 (36.0,43.9)	75.6 (72.1,78.8)	143.5 (141.8,145.2)	0.7 (0.3,1.6)	26.7 (26.3,27.1)
• 70–79	44.5 (37.8,51.4)	69.8 (63.1,75.7)	147.6 (144.8,150.4)	1.5 (0.7,3.1)	25.1 (24.5,25.7)
• ≥ 80	34.0 (24.4,45.0)	70.4 (59.9,79.2)	150.4 (145.4,155.4)	4.3 (1.4,12.1)	22.6 (21.6,23.5)

BP = blood pressure, BMI = body mass index

The ordinal regression (Table 3) showed that factors were associated with moderate and high 10-year CVD risks when compared to low risk. The factors associated with these risks were Malay ethnicity (OR 0.76, 95%CI 0.63–0.92, p = 0.004), unmarried status (OR 1.55, 95%CI 1.22–1.97, p<0.001) and physically inactive (OR 0.72, 95%CI 0.55–0.95, p = 0.020).

**Table 3:** Ordinal regression on factors associated with cardiovascular risk using non-laboratory based FRS.

	Estimates (SE)	Cumulative odds ratio	95% confidence interval	P value
**FRS**				
• 10-year risk of >20%	1.01 (0.220)	2.74	1.78–4.22	<0.001*
• 10-year risk of 10–20%	2.63 (0.225)	13.82	8.88–21.5	<0.001*
• 10-year risk of <10%		Ref		
**Ethnicity**				
• Malay	-0.23 (0.096)	0.76	0.63–0.92	0.004*
• Non-Malay		Ref		
**Marital status**				
• Unmarried	0.44 (0.121)	1.55	1.22–1.97	<0.001*
• Married		Ref		
**Highest education level**				
• No formal	0.03 (0.149)	1.10	0.82–1.47	9.533
• Primary	-0.04 (0.120)	0.96	0.76–1.21	0.728
• Secondary or higher		Ref		
**Monthly household income**	0.15 (0.116)	1.16	0.93–1.46	0.197
**Waist circumference**				
• Abnormal	-0.06 (0.110)	0.57	0.76–1.16	0.567
• Normal		Ref		
**Physical activity**				
Inactive	-0.32 (0.139)	0.72	0.55–0.95	0.020*
Minimally active	0.09 (0.145)	1.09	0.82–1.45	0.534
HEPA active		Ref		

FRS = framingham risk score, Ref = reference group, SE = standard error,

*p<0.05 = statistical significance

Chi-square = 4161.74; p = 0.321; Nagelkerke R^2^ = 0.011

## Discussion

Our study found two thirds of community dwelling older people had hypertension and dyslipidemia and almost half of them had diabetes. The women with hypertension in our study had higher mean systolic BP compared to men and more women had dyslipidemia compared to men. The mean BMI for both older men and women were in the range of overweight and were significantly higher among those aged 60–69 years. The factors associated with moderate and high 10-year CVD risk were Malay ethnicity, unmarried status and physically inactive, when compared to participants with low 10-year CVD risk.

Our study found the prevalence of hypertension among the older people was comparable with other Asian studies, which ranged between 50% and 70% [20, 21]. Our study found women had higher levels of SBP than men, and systolic BP increases with age similar to previous studies [20, 21]. It has shown that menopause in women was associated with higher blood pressure independent of age and BMI [22].

The prevalence of dyslipidaemia in our study population was higher at 65% when compared to other Asian studies among older people, which ranged from 37–44% [9, 20]. In addition, the mean BMI in our study were in the range of overweight for both men and women in those aged less than 70 years, of whom the prevalence of dyslipidemia was the highest across the different age groups. Up to the age of 70 years the muscle mass decreases and the fat mass increases [23]. Hence, this could explain the higher BMI in the age group less than 70 years.

About 40% of our study participants had diabetes. Similarly, the previous NHMS 2011 showed the prevalence of diabetes was lower in the age group of more than 75 year [24]. The incidence of diabetes increases with age up to the age of 65 years and after the age of 65 years, both the incidence and prevalence levels off [25].

The prevalence of current smokers among the older people has increased from 11.9% in the NHMS 2011 to 14.8% in the present study [26]. Similar to the earlier findings, more men than women were current smokers and the prevalence decreased with advancing age. In Malaysia, current smokers among older people was common and most were of Malay and other Bumiputera ethnicities. In the present study, higher proportion of participants were of Malay ethnicity, which could explain the increase in the prevalence.

The Malay ethnicity was found to be associated with moderate and high 10-year CVD risk when compared to participants with low 10-year CVD risk. We are not able to make comparisons with other studies as this is the first study that evaluated CVD risk among older people that included the Malay ethnicity. However, the possible reasons could be that other ethnic groups were found to attain better target control for the CVD risk factors such as blood pressure, glucose level and cholesterol levels, when compared to Malays [27].

Our study also found that unmarried status was associated with moderate 10-year CVD risk when compared to participants with low 10-year CVD risk. A previous study that evaluated CVD risk among older people in China found that unmarried status was associated with coronary artery disease events in men [28]. A previous review suggested that family and social support plays an important role in the adherence of diabetes management to achieve control [29]. It is possible that unmarried people may have less family/social support, which was not assessed in the present study.

Unlike in previous study by Li et. al.(2011), physical inactivity was associated with higher CVD risk among older people with T2DM in our study [10]. Also, majority of the older people were physically inactive, of which health promotion to engage in physical activity in older age is needed.

There were no significant associations between moderate and high 10-year CVD risk and other factors when compared to participants with low 10-year CVD risk. FRS underestimates CHD risk in older people, particularly in women [30]. Re-estimated risk functions using these factors improve accurate estimation of absolute risk. The actual risk prediction with FRS might perform less well in older people compared to middle-aged adults, and some traditional risk factors have weaker associations with CHD risk in the elderly; for example, total and LDL-cholesterol are strong cardiovascular risk factors in middle-aged but not in older people.

Almost three quarter of older people had a FRS high 10-year CVD risk and the prevalence of hypertension, dyslipidaemia and diabetes are high among the community dwelling older people in Malaysia. Initiation of treatment and individualised target control is imperative. Controlling CVD risk factors among older people do improve outcomes with greatest attention in reducing overall CVD risks. Therefore, this study provides insight on the need for future study to evaluate preventive strategies to improve the older people’s health in order to promote healthy and productive ageing.

## Supporting information

S1 File (PDF)

S2 File (PDF)
