# Supplementary material for: Cardiovascular disease risk factors among older people: Data from the National Health and Morbidity Survey 2015
Source: PLoS One. 2020 Oct 21;15(10):e0240826. doi: 10.1371/journal.pone.0240826 (PMC7577487; doi:10.1371/journal.pone.0240826)
Supplement: S1 File — (PDF) [file pone.0240826.s001.pdf]

JAWATANKUASA ETIKA & PENYELIDIKAN PERUBATAN
(Medical Research & Ethics Committee)
KEMENTERIAN KESIHATAN MALAYSIA
d/a Institut Pengurusan Kesihatan
Jalan Rumah Sakit, Bangsar **Tel : 03 2282 0491**
59000 Kuala Lumpur **Faks : 03 2282 8072 / 03 2282 0015**

Ref: (II) dlm.KKM/NIHSEC/ P14-1064
Date : 18 Februari 2015

NMRR-13-1214-14948
Molecular Autopsy of Sudden Unexplained Death Among Young Adults.

Principle Investigator : Dr Muhammad Fadhli Mohd Yusoff
Institut Kesihatan Umum

Documents received and reviewed with reference to the above study:

1. Study Proposal, version 01 dated 30-09-2014
2. Patient information sheet (English) & Informed Consent Form (English) version 02 dated 03-02-2015
3. Patient information sheet (Malay) & Informed Consent Form (Malay) version 02 dated
4. Questionairre 01 dated 30-09-2014
5. Investigator's CV , IAHOD, GCP
    - Dr Abdul Aiman bin Abd Ghani
    - Adilius Manual
    - Dr Ami Fazlin Syed Mohamed
    - Dr Fauziah binti Nordin
    - Hamizatul Akmal Binti Abd Hamid
    - Hasimah Ismail
    - Helen Tee Guat Hiong
    - Dr Jasvindar Kaur
    - Kamarul Zaman bin Salleh
    - Lim Kuang Hock
    - Mohamad Naim bin Mohamad Rasidi
    - Dr Mohd Azahadi Omar
    - Dr Noor Ani binti Ahmad
    - Dr Noor Safiza Binti Mohamad Nor
    - Nor Azian bt Mohd Zaki
    - Norrafizah Bt Jaafar
    - Rashidah Dato' Ambak
    - Dr Tahir Aris

The Medical Research & Ethics Committee, Ministry of Health Malaysia operates in accordance to the International Conference of Harmonization Good Clinical Practice Guidelines.

Please be informed that this approval is only valid until **18 Februari 2016**. To renew the approval, a completed *'Continuing Review Form'* should to be submitted to MREC at least 2 months before the expiry for the extension of the approval. You are also required to submit a study completion report upon completion of this study and report on all serious and unexpected adverse events to the Medical Research & Ethics Committee if relevant .These forms can be downloaded from the MREC web site (http://www.nih.gov.my/mrec).

Decision by Medical Research & Ethics Committee:
(√) Approved
(   ) Disapproved

Date of Decision: **18 Februari** 2015

(DATO' DR CHANG KIAN MENG)
Chairman
Medical Research & Ethics Committee
Ministry of Health Malaysia
