# Supplementary material for: Cardiovascular disease risk factors among older people: Data from the National Health and Morbidity Survey 2015
Source: PLoS One. 2020 Oct 21;15(10):e0240826. doi: 10.1371/journal.pone.0240826 (PMC7577487; doi:10.1371/journal.pone.0240826)
Supplement: S2 File — (PDF) [file pone.0240826.s002.pdf]

INSTITUT KESIHATAN NEGARA
NATIONAL INSTITUTES OF HEALTH (NIH)
KEMENTERIAN KESIHATAN MALAYSIA
MINISTRY OF HEALTH
PEJABAT PENGURUS NIH
Kompleks Institut Kesihatan Negara
No.1, Jalan Setia Murni U13/52
Seksyen U13 Setia Alam
40170 SHAH ALAM, SELANGOR

Telefon :603-33628888
:603-33628206
Faksimili :603-33628106
http://www.nih.gov.my

Ruj.Kami : KKM.NIHSEC.800-4/4/1 Jld. 65(43)
Tarikh : 11 Februari 2019

Pengarah
Institut Kesihatan Umum

Tuan,

KELULUSAN UNTUK MENERBITKAN ARTIKEL

Dengan hormatnya saya merujuk perkara di atas.

2. Sukacita dimaklumkan bahawa Ketua Pengarah Kesihatan Malaysia telah **meluluskan** permohonan **Dr Rajini Sooryanarayana** dari **Pusat Penyelidikan Kesihatan Keluarga** yang bertajuk:

" *Cardiovascular Disease Risk Factors among Older People: Data from The National Health and Morbidity Survey 2015* " untuk diterbitkan dalam jurnal *PLOS One.*

3. Sehubungan dengan itu sesalinan penerbitan tersebut hendaklah dikemukakan kepada Unit Komunikasi & Sebaran Saintifik, Institut Kesihatan Negara sebaik sahaja ianya diterbitkan.

Sekian, terima kasih.

"BERKHIDMAT UNTUK NEGARA"

Saya yang menjalankan amanah,

(MOHD IDRIS BIN OMAR)
Ketua Unit Komunikasi & Sebaran Saintifik
b.p Pengurus NIH
Institut Kesihatan Negara (NIH)
Kementerian Kesihatan Malaysia

Translation for the Permission Letter to Publish

Director,
Public Health Institute

Sir,

APPROVAL TO PUBLISH AN ARTICLE

With all due respect the above matter is referred.

2. We are pleased to inform that the Director General of Health Malaysia have approved the application by Dr Rajini Sooryanarayana from Centre of Family Health Research entitled:

"Cardiovascular disease risk factors among older people: Data from the National Health and Morbidity Survey 2015" to be published in the PLOS One journal.

3. Therefore, a copy of the publication is to be submitted to the Unit of Communication & Scientific Dissemination, National Institute of Health) secretariat upon its being published.

Thank you.

"Serving the Nation"

I who has been entrusted,

MOHD IDRUS B OMAR
Head, Unit of Communication & Scientific Dissemination
o/b Director NIH
National Institute of Health
Ministry of Health Malaysia
